# Aging-simulation experience: impact on health professionals’ social representations

**DOI:** 10.1186/s12877-019-1409-3

**Published:** 2020-01-22

**Authors:** Julie Giner Perot, Witold Jarzebowski, Carmelo Lafuente-Lafuente, Cyril Crozet, Joël Belmin

**Affiliations:** 1 Service de gériatrie du Pr Belmin, Hôpital Charles Foix, 94200 Ivry-sur-Seine, France; 2 Laboratoire Éducations et Pratiques de Santé, Université Paris 13, Bobigny, France; 3 Centre hospitalier de Bastia, Bastia, France; 4 Faculté de médecine, Sorbonne Université, Paris, France

**Keywords:** Aging suit, Age-related limitations, Attitudes, Social representations

## Abstract

**Background:**

Health professionals working with older persons are not sufficiently aware of the sensory and functional difficulties experienced by older patients. Innovative educational activities, such as the aging-simulation experience, can facilitate this awareness. This study describes the effects of an aging-simulation experience on health professionals’ representations towards age-related limitations.

**Methods:**

306 health professionals, enrolled in university training in geriatrics/gerontology in the 2015–2016 and 2016–2017 academic years, experienced an aging-simulation session wearing a special suit according to a predefined scenario. Before and after the aging-simulation experience, participants completed free association tests, with the inductive words vision, hearing, movement, fine dexterity and balance. Semantic categories were created from participants’ free evocations using a correspondence table manually produced in Excel 2013 for Windows (Microsoft Corporation, Redmond, Washington). Moreover, participants’ opinions on difficulties experienced by older people in relation to age-related limitations were studied using Likert scale questions.

**Results:**

In total, 3060 free evocations were collected, and ten semantic categories were created. These categories were composed of participants’ geriatric knowledge, about age-related limitations, and participants’ feelings, about the experience of these limitations. These two aspects were impacted by the aging-simulation experience. Moreover, changes observed resulted in a better consideration of difficulties associated with age-related limitations.

**Conclusions:**

The aging-simulation experience is an effective educational tool to raise awareness among health professionals of age-related difficulties. This sensory activity allows health professionals to put themselves in the shoes of older patients and to feel age-related difficulties.

## Background

With the aging process, age-related functional and sensory disabilities mainly affect people aged 70 and over. Decreased visual and aural acuity, difficulty with mobility, decreased fine dexterity and loss of balance are the principal manifestations of these limitations. Thus, among people aged 80 and over, 26.7% have three or more physical limitations, 6.6% have two physical limitations and 9.6% have one physical limitation [11]. As a result of these disabilities, many older people can no longer live alone in their homes and require appropriate care.

Older patients represent an important part of the population in care services [2, 9, 15]. Due to the prevalence of age-related limitations, health professionals may develop a disabling vision of the aged body. Thus, age-discriminating behaviors were observed in various medical disciplines. The only age variable can influence medical decisions, particularly for invasive treatments, such as surgery, resuscitation or oncogeriatrics [1, 5]. Moreover, negative stereotypes of aging in society have increased continuously since the end of the nineteenth century [17]. Thus, health professionals may be inclined to consider age-related functional and sensory limitations as normal and not affecting the quality of life of older persons.

Changing health professionals’ viewpoint contributes to improve care for older persons. Lifelong learning plays an important role in enhancing representations toward older patients. Simulation-based medical teaching and learning has significantly expanded in recent years. The impact of procedural simulation techniques, on quality of care, in obstetrics [7] and emergency care [19, 25, 26], has been widely demonstrated. However, in geriatrics/gerontology, aging-simulation experience was not sufficiently studied.

The aging-simulation experience is an innovative pedagogical device allowing health students and professionals to experience the functional and sensory limitations associated with aging. This sensory activity improves health students’ empathetic attitudes towards older adults [4, 16, 18, 29] and generates positive attitudes among health professionals [8].

In this study, we deeply analyze the effects of the aging-simulation experience by focusing on its impact on health professionals’ representations towards age-related limitations. To our knowledge, this approach has never been realized until now.

## Methods

This quantitative study constituted the first part of a sequential explanatory research design (mixed methods); with a qualitative study, in the process of being published, compounding the second part. These two studies were included in a research program examining the impact of an aging-simulation experience on representations, attitudes and care practices towards older persons.

### Participants

We included healthcare professionals working in geriatrics. These professionals undertook geriatrics/gerontological teaching over two academic years (2015–2016 and 2016–2017). We excluded the participants who had already realized the sensory activity, those with back pain, and pregnant women.

### Study design

We conducted a before/after study asking participants to complete a questionnaire on social representations towards age-related limitations, before and after the sensory activity. This experience was conducted in three phases. First of all, we informed participants about the contents of the workshop and the trainer reminded them of the rules of this educational activity (respect for others, mutual listening, mutual help). During this step, participants completed the pre-test questionnaire. Secondly, we conducted the sensory activity. Each participant carried the aging suit for an average of 15 min and performed different actions according to a defined scenario: going up and down stairs, lying down on the floor and getting up, sitting and getting up from a chair, drinking a glass of water and eating. Thirdly, participants completed the post-test questionnaire at the end of the sensory activity. Finally, we held a debriefing allowing participants to share experiences and emotions generated by the simulation activity.

### Questionnaire

Our questionnaire was constituted by five free association tests structured around five inductive words: vision, hearing, movement, fine dexterity and balance of older persons. The five inductive words were chosen because of the high prevalence of limitations affecting these functions. The five free association tests were worded as follows: “Could you indicate three words that you think best represent the vision of older people?” The pattern of these questions was the same for all the five inductive words vision/hearing/movement/fine dexterity and balance.

To complete these free association tests, participants’ opinions on the difficulties experienced by older people, in relation to age-related limitations, were studied before and after the simulation using five Likert scale questions. These questions were worded as follows: “In your opinion, do older people experience difficulties related to visual decline in daily life? Yes severe/yes significant/yes moderate/yes mild/none/no answer”. The pattern of these questions was the same for the difficulties related to visual decline/to hearing decline/to movement disorders/ to alteration of fine dexterity and to balance disorders. To avoid restricting speech, we conducted free association tests before Likert scale questions.

### Age-simulation suit

Several elements forming the GERT® age-simulation suit (Wolfgang Moll, Germany) were used in our study: overshoes reducing perception of the ground, knee and elbow pads limiting movement, weights on wrists and ankles reproducing muscle loss and making movements more difficult and less precise, a ballasted plastron (about 5 Kilograms) causing an arched posture and slowing movement, a cervical collar limiting neck mobility, a headset and glasses respectively simulating the presbycusis and the presbyopia, gloves limiting finger mobility and simulating loss of sensitivity.

### Data processing

We transferred the participants’ free evocations^18^ into Excel 2013 for Windows (Microsoft Corporation, Redmond, Washington). We created semantic categories from these free evocations, using a correspondence table manually produced in Excel 2013. This table, enriched progressively, was the result of a consensus between two researchers. Using Excel 2013, we calculated the number and percentage of participants who cited each category before and after the aging-simulation experience. We retained the categories cited by at least 10% of participants before and after the experience. The categories cited by less than 10% of participants were not considered in further data analyses. For the analysis of the Likert scale questions, the number and percentage of each type of answer *yes severe/yes significant/yes moderate/yes mild/none/no answer,* were calculated in Excel 2013 before and after the aging-simulation experience.

### Statistical analysis

We applied the McNemar test and the odds ratio calculation to compare participants’ free evocations before and after the sensory activity. We used the chi-square test to compare participants’ answers to the Likert scale questions. We performed these statistical tests using XLSTAT 2017.6 software for Windows (Microsoft Corporation, Redmond, Washington).

### Ethical issues

We informed all the participants about the study and we received their agreement to participate. We were careful to limit risk to participants by excluding pregnant women and individuals with back pain. In addition, we accompanied participants during the simulation experience to avoid any accidents. Data collected were handled and stored in accordance with the tenets of the Declaration of Helsinki (2008). According to the Jardé Law on research involving the human person, whose implementing decree came into force on 18 November 2016, such research does not require ethical approval. Verbal informed consent, sufficient for this type of research, according to current legislation, was obtained from all participants.

## Results

We proposed the aging-simulation experience to 325 health professionals. Among them, 19 (6%) were excluded of the study due to eligibility criteria: 7 (2%) participants had already realized the sensory activity, 10 (3%) participants had back pain and 2 (1%) participants were pregnant women. Among the 306 health professionals included in the study, 69% (*n* = 211) were female, 31% (*n* = 95) were male and the average age was 42 (18–68) years. Participants were working as physicians (*n* = 157, 51%), nurses (*n* = 44, 14%), occupational therapists (*n* = 18, 6%), psychomotor therapists (*n* = 17, 5%), health managers (*n* = 15, 5%), psychologists (n = 15, 5%), pharmacists (*n* = 12, 4%), physical therapists (*n* = 10, 3%), osteopaths (*n* = 8, 3%), orderlies (*n* = 5, 2%) and dieticians (n = 5, 2%). In parallel with their professional activity, participants were enrolled in geriatrics/gerontology training including a university degree (*n* = 162, 53%), a master’s degree (*n* = 75, 24%) and a short two-day training session (*n* = 54, 18%). Fifteen (5%) participants did not mention the training taken.

### Participants’ representations towards age-related limitations and impact of the aging-simulation

In total, we collected 3060 free evocations. We classified these free evocations into ten semantic categories: *decreased hearing, decreased vision, stiffness, slowness, loss of balance, fall, clumsiness, negative emotions, loneliness* and *difficulties*. We noted that these categories focused on two aspects. The first aspect concerned participants’ geriatric knowledge about age-related functional and sensory limitations and included seven categories: *decreased hearing, decreased vision, stiffness, slowness, loss of balance, fall* and *clumsiness*. The second aspect concerned participants’ feelings relating to the experience of these limitations and included three categories: *negative emotions, loneliness* and *difficulties*.

We observed statistically significant changes in the categories quoted by participants before and after the aging-simulation experience. First of all, looking at the results for the whole of the five free association tests (Table 1), we noted that fewer participants cited the categories *decreased hearing* (from 268 (88%) to 242 (79%), *P* = .002) and *slowness* (from 232 (76%) to 190 (62%), *P* < .001) after the simulation experience than before. Conversely, after the experience, more participants cited the categories *stiffness* (from 34 (11%) to 52 (17%), *P* = .02), *loneliness* (from 35 (11%) to 76 (25%), *P* < .001) and *difficulties* (from 129 (42%) to 183 (60%), P < .001). Secondly, looking independently at each free association test (Table 2), we noted several changes: for the free association test 1 (inductive word “Vision”), the number of participants citing the category *decreased vision* (from 272 (89%) to 280 (92%), OR 1.03 (0.60–1.76)) increased after the sensory activity; for the free association test 2 (inductive word “Hearing”), the number of participants citing the category *decreased hearing* (from 265 (87%) to 240 (78%), OR 0.91 (0.59–1.39)) declined after the sensory activity and this number increased for the category *loneliness* (from 32 (10%) to 76 (25%), OR 2.38 (1.52–3.72)); for the free association test 3 (inductive word “Movement”), the number of participants citing the categories *slowness* (from 217 (71%) to 183 (60%), OR 0.84 (0.60–1.18)), *loss of balance* (from 46 (15%) to 39 (13%), OR 0.85 (0.54–1.34)) and *negative emotions* (from 73 (24%) to 62 (20%), OR 0.85 (0.58–1.25)) declined after the sensory activity and this number increased for the category *difficulties* (from 54 (18%) to 105 (34%), OR 1.94 (1.33–2.83)); for the free association test 4 (inductive word “Fine dexterity”), the number of participants citing the categories *slowness* (from 47 (15%) to 54 (18%), OR 1.15 (0.75–1.76)), *negative emotions* (from 39 (13%) to 46 (15%), OR 1.18 (0.74–1.87)) and *difficulties* (from 61 (20%) to 104 (34%), OR 1.7 (1.18–2.46)) increased after the sensory activity; and, for the free association test 5 (inductive word “Balance”), the number of participants citing the categories *loss of balance* (from 241 (79%) to 250 (82%), OR 1.04 (0.7–1.55)) and *fall* (from 50 (16%) to 65 (21%), OR 1.3 (0.86–1.96)) increased after the sensory activity.

**Table 1:** Number and percentage of participants (2015–2016 and 2016–2017 academic years) citing the different semantic categories before and after the simulation experience, for all free association tests

Categories	Participants before (*n* = 306)	Participants after (*n* = 306)	*P* value
Decreased hearing	268 (88%)	242 (79%)	.002
Decreased vision	268 (88%)	282 (92%)	.72
Stiffness	34 (11%)	52 (17%)	.02
Slowness	232 (76%)	190 (62%)	< .001
Loss of balance	246 (80%)	251 (82%)	.60
Fall	66 (22%)	62 (20%)	.64
Clumsiness	130 (42%)	137 (45%)	.55
Negative emotions	127 (42%)	144 (47%)	.11
Loneliness	35 (11%)	76 (25%)	< .001
Difficulties	129 (42%)	183 (60%)	< .001

We expressed results as numbers and percentages in brackets, except for *p* values

**Table 2:** Number and percentage of participants (2015–2016 and 2016–2017 academic years) citing the different semantic categories, before and after the simulation experience, for each free association test

Inductive words and semantic categories	Participants Before (*n* = 306)	Participants After (*n* = 306)	OR (95% CI)
« Vision » (Free association of words test 1)			
Decreased vision	272(89)	280(92)	1.03(0.60–1.76)
« Hearing » (Free association of words test 2)			
Decreased hearing	265(87)	240(78)	0.91(0.59–1.39)
Loneliness	32(10)	76(25)	2.38(1.52–3.72)
« Movement » (Free association of words test 3)			
Slowness	217(71)	183(60)	0.84(0.60–1.18)
Loss of balance	46(15)	39(13)	0.85(0.54–1.34)
Negative emotions	73(24)	62(20)	0.85(0.58–1.25)
Difficulties	54(18)	105(34)	1.94(1.33–2.83)
« Fine dexterity » (Free association of words test 4)			
Slowness	47(15)	54(18)	1.15(0.75–1.76)
Clumsiness	131(43)	133(43)	1.02(0.74–1.40)
Negative emotions	39(13)	46(15)	1.18(0.74–1.87)
Difficulties	61(20)	104(34)	1.7(1.18–2.46)
« Balance » (Free association of words test 5)			
Loss of balance	241(79)	250(82)	1.04(0.7–1.55)
Fall	50(16)	65(21)	1.3(0.86–1.96)

*Abbreviations*: *Part*. indicates participants, *OR* odds ratio, *CI* confidence interval

We expressed results as numbers and percentages in brackets, except for odds ratios

Only categories stated by at least 10% of the participants, before and after the simulation experience, were retained for data analyses

### Participants’ opinions on age-related difficulties and impact of the aging-simulation

**Fig. 1:**
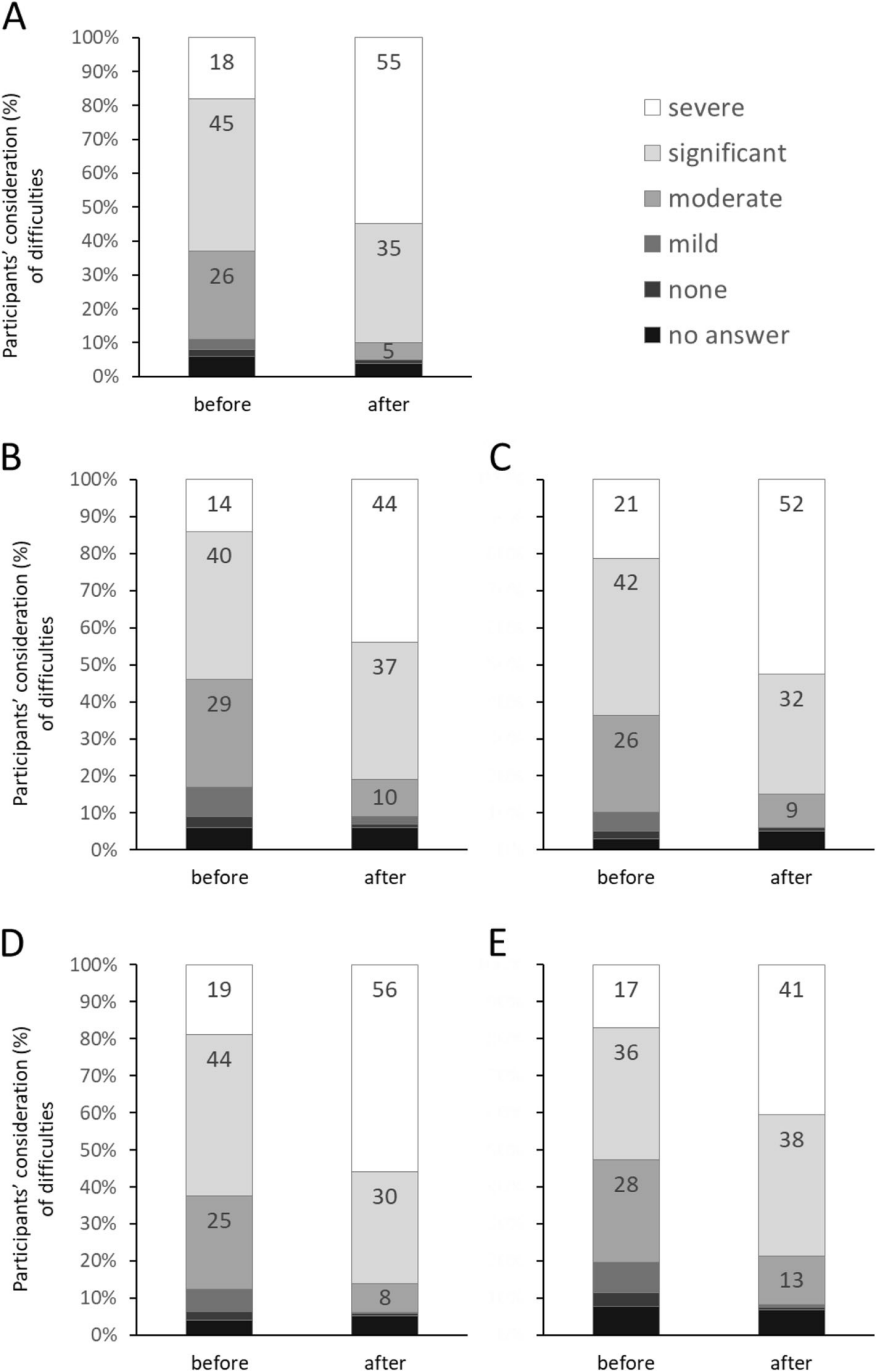
Participants’ opinions (n = 306), before and after the aging-simulation experience, on difficulties experienced by older people in relation to age-related limitations (panel **a**: movement disorders; panel **b**: visual decline; panel **c**: alteration of fine dexterity; panel **d**: hearing decline; panel **e**: balance disorders)

Participants’ opinions on difficulties experienced by older people were impacted by the aging-simulation experience. Indeed, whatever the limitation questioned (visual decline/hearing decline/movement disorders/alteration of fine dexterity/balance disorders), we observed large differences in the distribution of participants’ answers pre- and post-sensory activity (Fig. 1). Particularly, the number of participants considering that older persons experience severe or significant difficulties related to movement disorders increased after the sensory activity (from *n* = 193 (63%) to *n* = 275 (90%), *P* < .001). The same pattern was observed for difficulties related to visual decline (from *n* = 165 (54%) to *n* = 248 (81%), *P* < .001), to hearing decline (from *n* = 193 (63%) to *n* = 263 (86%), *P* < .001), to alteration of fine dexterity (from *n* = 193 (63%) to *n* = 257 (84%), *P* < .001) and to balance disorders (from *n* = 162 (53%) to *n* = 242 (79%), P < .001). Thus, participants considered age-related difficulties to be more important after the sensory activity than before.

## Discussion

In this study we were interested in the impact of an aging-simulation experience on health professionals’ representations towards age-related limitations. We described that these representations were constituted by participants’ feelings and by participants’ geriatric knowledge about age-related limitations. We observed that these two aspects were impacted by the aging-simulation experience. Furthermore, after the sensory experience, participants’ opinions were oriented on a better understanding of difficulties experienced by older people. It is interesting to observe that, among a group of health professionals working with older patients, the aging-simulation experience impacted feelings and knowledge. We hypothesize that the strong emotional power [28] of this educational device, contributed to change the representations of this group of professionals experienced in geriatrics.

An original point of our study was using free association tests to explore representations of age-related limitations in geriatrics, these tests allowing a satisfactory investigation. The creation of a correspondence table, between participants’ free evocations and semantic categories, permitted to precisely describe these representations. The approach developed in this study is original and complementary to previous research focusing on the impact of this sensory experience on health students and professionals’ attitudes towards older persons. Indeed, earlier studies showed that the aging-simulation experience raised awareness of the importance of environmental adaptation to limit disabilities related to functional and sensory limitations [23] and improved medical students’ positive attitudes and empathy towards older patients [16, 18, 23, 29]. Similar results, regarding positive attitudes and empathy, were found among pharmacy students [4] and among students in dietetics and physiotherapy [6]. Moreover, among health professionals working in geriatrics, the simulation experience promoted respect for dignity in care [24] and also improved positive attitudes towards older patients [8, 14]. Our study, by focusing on the impact of this sensory activity on health professionals’ representations, complements these results and supports the hypothesis that the feeling of the age-related limitations by the health professionals has a significant impact on their thinking.

However, this study had several notable limitations. Firstly, there is a selection bias due to the choice of our population of both health professionals and students. Indeed, the dual learner/health professional profile was relevant for our research because it allowed an easy recruitment and possibility to follow participants over time. However, persons enrolled in training have characteristics that do not attribute to the whole group of health professionals, such as reflexive posture, analytics abilities and motivation to learn [3]. Moreover, it is possible that participants initially had a particular conception of older persons due to their practice in geriatric services. For these two reasons, we cannot generalize the results to the entire population of health professionals. Secondly, there is a social desirability bias [10, 12] related to the use of a discursive method (verbal association task) to collect representations. This bias may have influenced participants’ responses. Thirdly, the level of fidelity of the aging-simulation suit is questionable because this suit did not simulate pain [13, 20] and dementia [21, 22] whose prevalence increases with aging. Nevertheless, this question regarding fidelity [27] of pedagogical simulation exists whatever the field concerned (e.g. health, aviation) and it is important to remember that, beyond the simulator’s fidelity level, authenticity of the learning environment has great importance in simulation experience.

## Conclusion

The investigation of social representations towards age-related limitations, located halfway between social representations of old age and social representations of health and disease, presents a real utility because it targets a large community of professionals and patients. For this work to be complete, an investigation into the long-term repercussions of the aging-simulation experience is required. Thus, our upcoming qualitative study, forming the second part of our sequential explanatory research design, will finalize this work.

## Data Availability

All the data generated and analyzed in this study are not publicly available due to the very large amount of literal data analyzed. These data are of course available on request from the corresponding author.
